# An assessment of pelvic binder placement on patients referred to the Irish national centre for pelvic and acetabular fractures

**DOI:** 10.1007/s11845-025-04262-2

**Published:** 2026-01-08

**Authors:** C. Timon, C. Kilkenny, K. O’Sullivan, A Bracken, J. Fournier, B. O’Daly

**Affiliations:** Tallaght University Hospital, Dublin, Ireland

**Keywords:** Pelvic trauma, Polytrauma, Binder, Pelvic ring

## Abstract

**Background:**

Pelvic binders are used to reduce the haemorrhage associated with pelvic ring injuries. Application at the level of the greater trochanters is required. We assessed the frequency of their use in patients with pelvic ring injuries and their positioning in patients being referred to the Irish national centre for treatment of pelvic & acetabular fractures.

**Aims/Methods:**

A retrospective review of our trauma database was performed to consecutively select 91 patients for study from May 2024 to May 2025 Patients with a pelvic ring injury defined by the Young and Burgess classification were included. Computed tomography was used to identify and measure pelvic binder placement.

**Results:**

22/91 (24%) had a binder placed. Of the total, 11/91 (12%) patients had satisfactory placement and 11/91 (12%) had unsatisfactory placement; 69/91 (76%) patients with a pelvic ring injury had no binder applied.

**Conclusion:**

This is the first study assessing pelvic binder placement in patients referred to the national pelvic and acetabular injury unit. Unsatisfactory positioning of the pelvic binder is a common problem and it was not used in a large proportion of patients with pelvic ring injuries. This demonstrates that there is a need for continuing education for teams dealing with major trauma.

## Introduction

Unstable pelvic ring injuries may lead to loss of life through haemorrhage. Bleeding most commonly occurs from the posterior pelvic venous plexus but may also arise from fracture ends, arterial sources and associated injuries [1]. Early appropriate management is important to prevent haemorrhage and its associated morbidity and mortality [2].

Pelvic binders or, more broadly, pelvic circumferential compression devices (PCCDs) are an important part of the management of pelvic ring injuries. They are available in both the pre-hospital and emergency department setting and are advantageous in their ability to be applied expediently without the need for an operating theatre or specialist surgical training. They encourage clot formation by stabilising the injury and reduce the size of the intrapelvic volume in which haemorrhage can accumulate [3].

Studies have shown that PCCDs are required to be placed at the level of the greater trochanters to function correctly. This position effectively stabilises the pelvic injury, carries physiological benefits for the patient, and is the best position in which to reduce a symphyseal diastasis [4]. Despite this knowledge and its incorporation into Advanced Trauma Life Support guidelines [4], a previous UK study has shown that only 49% of binders were positioned correctly [5], and a study in the US has demonstrated that PCCDs were absent in 53% of unstable pelvic injuries [6].

The aims of this study were to assess the frequency of application of pelvic binders in patients referred to the Irish national centre for treatment of pelvic & acetabular fractures with a pelvic ring injury and to assess the position of the pelvic binder in patients presenting as a major trauma, irrespective of whether a pelvic ring injury was found.

## Methods

### Patient selection

From the pelvic and acetabular fracture database at Tallaght University Hospital, we selected 91 consecutive patients with a pelvic ring injury for retrospective review. These were patients coded as sustaining either a pelvic ring injury who presented between 01/05/2024 and 01/05/2025. The electronic case records of these patients were used to identify those with either an injury to the pelvic ring defined by the Young and Burgess classification [7] (lateral compression I–III, anterior–posterior compression I–III or vertical shear) or any patient with a pelvic binder applied, irrespective of whether a pelvic ring injury was later found to be present.

### Assessment of binder position and presence

The computed tomography (CT) scans for these patients were reviewed by 2 independent observers to identify the presence of a pelvic binder The referral case notes were used to confirm that no alternative PCCD was applied (including bed sheets) that could not be visualised radiologically.

**Fig. 1:**
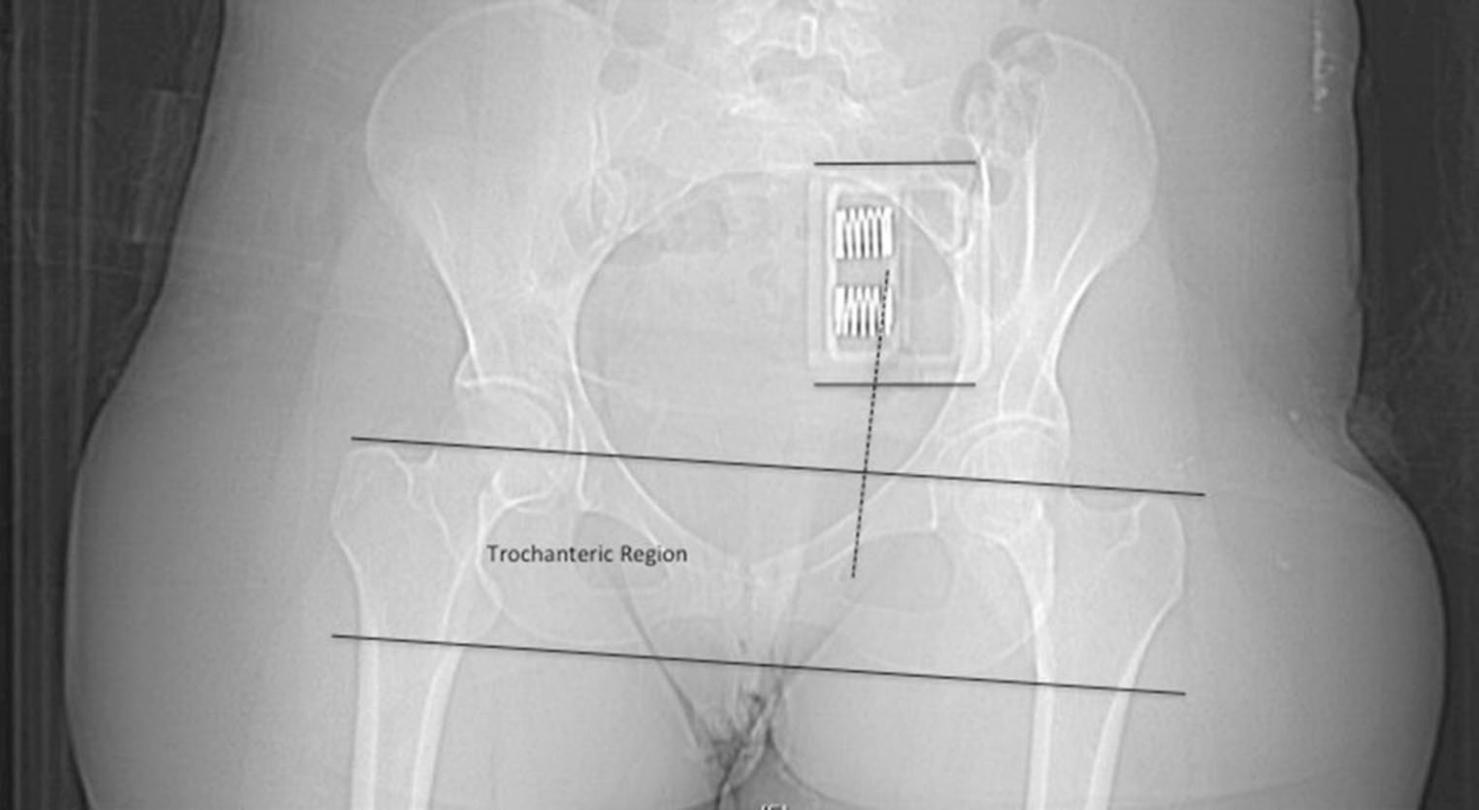
Measuring the position of the pelvic binder

The position of the binder was quantified by finding the mid-point between the greater and lesser trochanters (mid-trochanteric point) and the midpoint of the binder buckle. The distance between these two points (dotted line, Fig. 1) was then measured at 90 degrees to the plane of the trochanters. In addition, if the midpoint of the buckle was within the intertrochanteric space (intertrochanteric region, Fig. 1), this was considered to be a satisfactory position. If it was outside this area it was considered to be high or low. Radiological assessments were performed electronically using the NIMIS radiological storage system.

## Results

### Patient group

Of the 91 patients 46 (51%) were male and 45 (49%) were female. Average age was 63.5 years (range 15–84 years). The mechanism of injury was silver trauma in 34/91 (37%), road traffic accidents in 25/91 (27%), falls from height in 25/91 (27%), crush injuries in 4/91 (5%) and other in 3/91 (4%).

### Classification of injuries

Pelvic ring injuries were classified as per the Young and Burgess classification [7]. 56/91 (61%) patients had an LC1 type injury, 22/91 (24%) had an LC2 type injury, 3/91 (3%) had an LC3 type injury. 3/91 (3%) had an APC1 type injury, 5/91 (6%) had an APC2 type injury and 2/91 (2%) had an APC3 type injury.

### Assessment of binder position

In the 22 patients with pelvic binders, 11 (50%) were in a satisfactory position. In 69 of 91 (76%%) patients with a pelvic ring injury, there was no attempt to apply any PCCD.

### Assessment of binder type by mechanism

Of the 34 patients with silver trauma i.e. low mechanism injuries 2/34 (6%) had a PCCD applied. Of the 57 patients who had a high energy mechanism of injury (fall from height, road traffic accident, crush injury), 20/57 (35%) had a PCCD applied.

### Assessment of binder position by stable/ unstable injury type

Of the 32patients with unstable pelvic ring injuries (LC2, LC3, APC 2, APC3), 15/32 (47%) had a PCCD applied, 8 (25%) of whom had the PCCD applied correctly. 17/32 (53%) with unstable pelvic ring injuries did not have a PCCD applied.

## Discussion

This is the first study in the published literature to date assessing pelvic binder placement in Irish trauma patients. Unsatisfactory placement of PCCDs and lack of timely recognition of a serious pelvic injury remain common problems in the management of pelvic trauma and are clearly demonstrated by our results.

Our results of pelvic binder positioning, in those with a binder placed, demonstrate incorrect placement in 50% of patients and are very similar to those of a previous studies. A lack of knowledge of where to apply the binder has previously been identified in a written survey of trauma units in the UK. There, a significant proportion of emergency medicine registrars (60%) and trauma and orthopaedic registrars (20%) did not identify the greater trochanters as the correct level of binder application [8]. These findings are concerning given that incorrect positioning reduces their efficacy [1, 3, 4, 6]. This is also despite the popularity of training such as the Advanced Trauma Life Support course and the European Trauma Course.

The NICE guidelines on complex fractures assessment and management state that if active bleeding is suspected from a pelvic fracture following blunt high energy trauma a purpose-made pelvic binder, or if it does not fit an improvised pelvic binder should be fitted [9]. Unfortunately our trauma records do not assess whether patients were haemodynamically unstable at time of assessment.

The index of suspicion for unstable pelvic injuries also remains low: 69 of 91 patients with a pelvic ring injury had no binder applied.

Vaidya’s US study demonstrated similar findings with an absence of a PCCD in 37% of patients with an unstable anterior–posterior compression or vertical shear injury [6]. Clinical diagnosis of a pelvic ring injury is difficult and external clues may be absent. “Springing” the pelvis is no longer recommended as it may disturb haematoma clotting and cause further bleeding and also has a poor sensitivity and specificity [10]. Given the potentially devastating consequences of an untreated pelvic fracture, we recommend that PCCDs should be used routinely in cases of high-energy trauma.

That being said, the application of PCCDs is not without its own complications. Iatrogenic skin necrosis and peroneal nerve palsies have been described in the literature [11]. A 2016 systematic review, however, revealed that these complications are confined to case reports [12]. Furthermore, hard cervical collars, which are routinely recommended in the management of major trauma, have been shown to carry a higher risk of skin breakdown of up to 6.8% [13]. No complications from PCCD application were identified in our study. The potential over-reduction of lateral compression injuries is an argument against widespread binder use, although it can be argued that this represents the binder performing its function and there is no significant evidence that harm is caused. Case reports do demonstrate the uncommon possibility that a binder can mask a potentially unstable pelvic injury on CT [14]. If clinical suspicion exists, we recommend that the binder be carefully loosened in an adequately resourced care setting and that “binder off” plain radiography be performed as soon as possible to exclude an unstable injury.

As CT was used to assess the binders, the study would not include patients with pelvic injuries with absent or incorrectly positioned binders who died from the time of injury to their assessment in hospital prior to any imaging.

One of the limitations of this study is that it is restricted only to patients referred to the Irish national treatment centre for pelvic and acetabular fractures, there is a small number of orthopaedic trauma units in Ireland that manage their own pelvic ring injuries. Paediatric patients were not studied as patients aged 16 and younger are not treated at this centre. Finally, a pelvic binder can lose position after its application, either over time or during transfer. However, by assessing its position on CT, it is reasonable to believe that any malposition of the binder should have been identified and rectified by the trauma team at the initial primary survey.

## Conclusions

Our results demonstrate that the application of PCCDs was unsatisfactory in the majority of patients referred to our centre. A PCCD was not applied in 76% of patients with a pelvic ring injury. This highlights a need for continuing education for paramedics, emergency department staff and orthopaedic teams dealing with patients who have suffered major trauma. We believe the potential benefits in the routine use of PCCDs outweighs the small risk of complications and recommend routine use of a PCCD in all patients with high-energy trauma and a suspicious mechanism.
